# Automatic Optimization of the Computation Graph in the Nengo Neural Network Simulator

**DOI:** 10.3389/fninf.2017.00033

**Published:** 2017-05-04

**Authors:** Jan Gosmann, Chris Eliasmith

**Affiliations:** Centre for Theoretical Neuroscience, University of WaterlooWaterloo, ON, Canada

**Keywords:** Nengo, computation graph, optimization, Python, OpenCL, neural engineering framework

## Abstract

One critical factor limiting the size of neural cognitive models is the time required to simulate such models. To reduce simulation time, specialized hardware is often used. However, such hardware can be costly, not readily available, or require specialized software implementations that are difficult to maintain. Here, we present an algorithm that optimizes the computational graph of the Nengo neural network simulator, allowing simulations to run more quickly on commodity hardware. This is achieved by merging identical operations into single operations and restructuring the accessed data in larger blocks of sequential memory. In this way, a time speed-up of up to 6.8 is obtained. While this does not beat the specialized OpenCL implementation of Nengo, this optimization is available on any platform that can run Python. In contrast, the OpenCL implementation supports fewer platforms and can be difficult to install.

## 1. Introduction

Computational modeling is an important part of neuroscience and cognitive science. It allows us to make sense of data, generate predictions, and explicitly test theories and our understanding of cognitive mechanisms. In this endeavor there is a need for increasingly large models, especially as more and more individual cognitive functions get unified within single models.

For example, Schmidt et al. (2015) presented a model of vision-related macaque cortex areas with 4 million spiking neurons requiring a supercomputer to simulate. Similarly, the largest functional brain model to date, Spaun (Eliasmith et al., 2012), consists of 2.5 million neurons that originally required 2 h to simulate a single second of simulation time. Even though current implementations of Spaun have improved the simulation speed significantly due to both better hardware and improved simulation software—Spaun still imposes a considerable computational burden. As well, newer versions of Spaun include approximately 4 million neurons. Thus, it remains important to reduce simulation times in order to accelerate the debugging and development of large-scale models. Similar challenges are faced by the machine learning community as they increase the scale of their models likewise.

One of the most common approaches to reduce the runtime of such neural network models is to switch to more specialized hardware. It is perhaps most common to use GPUs (instead of CPUs) that are optimized for parallel algebraic operations. There is also ongoing work that is building even more specialized hardware for simulating spiking neural networks in real-time with minimal energy consumption (Furber, 2016), for example SpiNNaker (Furber et al., 2014) and Neurogrid (Benjamin et al., 2014).

While specialized hardware platforms are quite effective, they are, unfortunately, not available to everyone. Many such platforms are still in heavy development, and SpiNNaker and Neurogrid are only available to selected researchers. While GPUs are more broadly available, they still result in an additional cost and can be difficult to maintain for specialized applications. Additional costs can range from hundreds to thousands of dollars for GPUs, up to several thousands of dollars for neuromorphic hardware, if it is available. In addition, many high-performance clusters still predominantly use CPUs because the effective utilization of a large number of GPUs within a cluster remains an area of active research (Kindratenko et al., 2009). As a result, many HPC platforms provide access to far more CPU resources than GPUs. Consequently, there are many instances where deploying dedicated hardware platforms is not a viable option.

In these cases, the remaining options rely on software optimizations. This usually involves a trade-off between performance and maintainability. The reasons for this trade-off are manifold. For example, highly optimized code often needs to be written in low-level languages that are less suited to express the programmer's intent, whereas high-level languages incur additional computational overheads. As such, most scientific software today is written in a high-level language like Python, and only the performance critical parts are implemented in a low-level language like C or Fortran. A number of software tools like F2PY (Peterson, 2009), SWIG, Boost.Python, Instant (Wilbers et al., 2012), and SciPy Weave aim to make this integration easier. A slightly different approach is taken by Cython (Behnel et al., 2011) that extends the Python programming language to allow type annotations which then enable Cython to compile the annotated parts to efficient C code.

Nevertheless, there are downsides to these generic approaches when applied to neural simulation. For instance, they all require a working C or Fortran compiler. This can be a major hurdle in installing a software package for many users. Moreover, switching to a low-level language does not guarantee speed improvements. For some programming constructs like for-loops there will be less overhead, but performance problems are often more related to the employed algorithms, data structures, and memory layouts. Therefore, it can be more effective to improve these aspects of the simulation in the high-level language before attempting to switch to a low-level language.

One of the most common recommendations for scientific code in languages like Python and Matlab is to use vectorized operations instead of for-loops (van der Walt et al., 2011). These will invoke optimized code (usually written in C or Fortran) to perform the operation instead of looping within the high-level language that incurs a high overhead. This can be taken to an extreme where different array variables with the same operations applied to them will be merged into a single array. This can reduce the number of loops further and puts all the data into a consecutive memory segment. This latter aspect will be beneficial to the performance because due to the locality of the memory access it can be optimized for better caching in the CPU and for better pre-fetching of the required data. In most circumstances, merging arrays in such a way can easily lead to confusing code because it breaks the mental mapping between a single variable name and a single set of data described by that name. Here, we present a method to do this merging hidden from the user to keep the mental mapping intact.

Many packages for the simulation of neural networks like Nengo, Theano, and Tensorflow (Bekolay et al., 2014; Abadi et al., 2015; Al-Rfou et al., 2016) construct a computational graph, an idea rooted in dataflow programming (Culler and Culler, 1986; Johnston et al., 2004). Most of the work in the field of dataflow programming is concerned with the parallel execution of computation steps (e.g., Reiter, 1968; Miller, 1973; Hendrickson and Leland, 1995). Unfortunately, Python's global interpreter lock (GIL) prevents efficient parallel execution in Nengo. Less work has been done on ensuring locality of accessed data (Kavi and Hurson, 1998), perhaps because it is more difficult in the general case, and highly dependent on the instruction set (Miller, 1973). In Nengo, however, we can benefit from some pre-imposed structure on accessed memory, and the usage of the same linear algebra operations on many data fragments.

Accordingly, we present an algorithm implemented within the Nengo neural network simulator for automatic merging of arrays and applied operations to improve performance by increasing the locality of memory access. We demonstrate considerable simulation speed improvements at the cost of a moderately increased build time. This algorithm is of special importance in Nengo in the context of the optimization method for representational accuracy presented by Gosmann and Eliasmith (2016). This prior optimization method applies to neurons involved in the representation of uniformly distributed high-dimensional unit vectors, which is common in large-scale models like Spaun. The method makes use of the fact that a subvector composed of *k* components of an *n*-dimensional unit vector with *k* < *n* will usually be smaller than unit length. Thus, by splitting up the representation of the *n*-dimensional vectors across multiple small neuron groups representing *k*-dimensional parts, each group of neurons can be optimized to represent a smaller range which translates to an improvement in representational accuracy. Unfortunately, this has the downside of increasing the simulation time in Nengo. The algorithm presented here counteracts this increase in simulation time and allows the simulation to combine accurate representation and fast simulation speeds.

We will first introduce the Nengo neural network simulator (Section 2), before explaining the optimization algorithm in Section 3. The results from testing it on models of different sizes are presented in Section 4 and discussed in Section 5.

## 2. The nengo neural network simulator

The Nengo neural network simulator (Bekolay et al., 2014) is based on the methods of the Neural Engineering Framework (NEF; Eliasmith and Anderson, 2003). The NEF proposes three principles to enable the construction of large-scale neural models:

1. *Representation* describes how groups of neurons can represent time-varying vector values with non-linear encoding and linear decoding.
2. *Transformation* describes how optimal connection weights between two groups of neurons can be determined to implement linear and non-linear functions.
3. *Dynamics* describes how dynamical systems can be realized in neurons with recurrent connections.

Nengo uses these NEF principles to convert a high-level model description written in Python into a spiking neural network. The model description is independent of the hardware platform. As a result, the same model can be run on different “backends” (or “simulators”) that target specific hardware. Different backends can be used to run the same model on different types of hardware. Here we are mainly concerned with the reference backend that targets commodity CPUs. We will also use the OpenCL backend called Nengo-OCL as additional comparison in benchmarks that can target either CPUs or GPUs.

Internally, the reference backend represents the neural network with so-called *signals* and *operators* that implicitly define the computational graph to be executed for every simulation time step. Nengo-OCL uses a similar implementation, although backends are free to adopt other implementational structures.

Signals define values that are read and written by the simulator. For each high-level object, like a group of neurons, usually several signals will be created. A signal defines aspects like the vector or matrix shape of the value, data type, and initial value. As such signals provide information retained in consecutive blocks of memory that have to be allocated for the simulation.

Operators define how signals are updated given the values of other signals. Operators are typically common linear algebra operations like an elementwise product or dot product, but more specific operators are used for non-linear operations (such as the neuron non-linearity). The general type of the operation defines the *operator type* (e.g., copy), irrespective of the actual signals available. For each accessed signal, operators have to declare whether they *set, increment, read*, or *update* the signal. These operations are defined as follows within a simulation time step:

1. At most one (optional) *set* operation that defines the value of a signal at the start of the simulation time step.
2. Any number of *increment* operations modifying the signal value.
3. Any number of *read* operations retrieving the signal value.
4. At most one *update* operation that sets the final value of the signal for the next time step.

This implicitly defines a computational dependency graph. For example, operators that increment a signal need to be executed before that signal is read. The Nengo simulator constructs this dependency graph *G* and performs a topological sort to obtain the order in which the operators are executed. (There can be more than one valid order.)

Some operators will only operate on parts of a signal. In this case a *(signal) view* is assigned to the operator instead of a signal. Signal views are similar to views of NumPy arrays. In most cases they behave like a normal signal and have the same attributes. However, they do not define a distinct block of memory to be used for the simulation, but map into the memory block defined by another signal called the *base* of the view. The start of the view data is defined by an *offset* relative to the start of the base; the end of the view data is given by the view *size* relative to the offset.

## 3. Algorithm for operator merging

**Figure 1:**
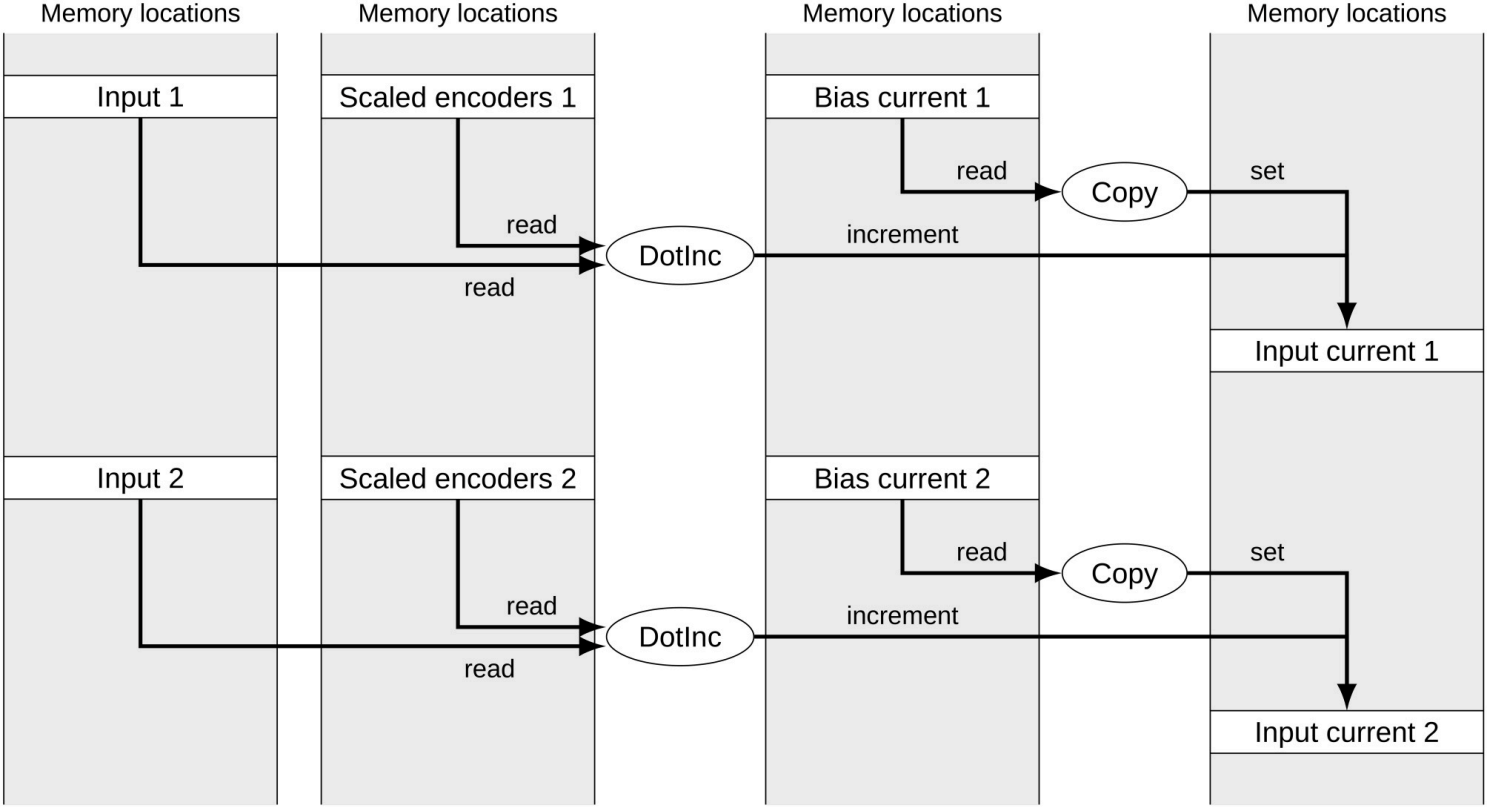
**Signals and operators to calculate the input current to two neural groups**. The gray boxes represent memory locations with allocated signals. Ellipses represent operators accessing and modifying these signals. Both sets of operators for the two neural groups access unrelated memory locations.

**Figure 2:**
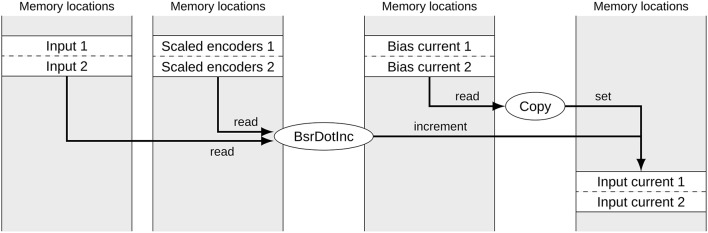
**Signals and operators to calculate the input current to two neural groups after merging**. The gray boxes represent memory locations with allocated signals. Ellipses represent operators accessing and modifying these signals. Due to the merging, the number of operators has been reduced to two and the memory access for corresponding signals is sequential.

Figure 1 shows a typical subset of operators and signals for calculating the input currents to two groups of neurons in a built Nengo model. Each of the signals corresponds to one block of memory. The memory location of data for each group of neurons can be considered random for our purposes. While the operators are calls to efficient NumPy functions, the reference backend loops over the operators in less efficient Python. The depicted part of the computational graph can be optimized by allocating data of the same type in sequential blocks and merging corresponding operators as shown in Figure 2. This allows the simulation to make better use of CPU caching and pre-fetching as more data is in sequential memory blocks, and the number of operators to loop over in Python has been reduced. In the following we discuss the detailed constraints of this optimization and how it can be performed automatically.

### 3.1. Merging of operators

Let us consider the *copy* operator as a simple example of how the merging of operators works. This operator copies the values from a *source* block of memory to a *destination* block. Given multiple copy operators, all the source and all the destination blocks can be concatenated to form sequential blocks of memory. Then just a single copy operator can be used for those concatenated blocks. While this requires allocating new memory blocks and moving data to the new memory location, there is a net benefit. This memory reorganization has only to be done once, but provides a speed-up in every simulation time step.

A more complicated example is merging the *DotInc* operator. This operator implements ***y***: = ***y*** + ***Ax***. To merge two of these operators into a single operator,

(1) $$\left[\begin{array}{c} y_{1}\\ y_{2} \end{array}\right]:=\;\left[\begin{array}{c} y_{1}\\ y_{2} \end{array}\right]+\left[\begin{array}{cc} A_{1} & 0\\ 0 & A_{2} \end{array}\right]\;[\begin{array}{c} x_{1}\\ x_{2} \end{array}]$$

has to be implemented. Unfortunately, a naive implementation of this would lead to a quadratic increase in memory consumption with the number of operators merged due to the structure of the ***A*** matrix. Because of that, the merged operator is replaced with a *BsrDotInc* operator that implements the same operation, but uses a block sparse matrix representation. This data structure does not need to represent the blocks of zeros in memory and thus does not incur any additional memory cost compared to the unmerged operators.

In more general terms, to allow the merging of two operators *o*_*i*_ and *o*_*j*_, it is sufficient that all of the following conditions are met:

1. Both operators need to be of the same type and that type has to support merging (e.g., copy operators can be merged, SimPyFunc operators implementing the execution of arbitrary user-provided Python code cannot be).
2. The execution of one operator must not depend on the other operator (e.g., DotInc depends on Copy in Figure 1). This is checked with the transitive closure on the dependency graph *G*, described in more detail in Section 3.6.
3. Each pair of signals (or signal views) $\left(s_{l}^{o_{i}},s_{l}^{o_{j}}\right)$ needs to allow merging. The exact conditions will be discussed in the next section.
4. The operator type might pose further requirements.

Note that a sequence of operators *o*_1_, …, *o*_*n*_ can be merged as a whole if all pairs (*o*_*i*_, *o*_*i*+1_) can be merged.

### 3.2. Merging of signals and signal views

Two (non-view) signals *s*_*x*_ and *s*_*y*_ can be merged into a single signal if two conditions are met: they have to use the same data type and their shapes have to agree on all axes except for the concatenation axis. To merge those signals, a new block of sequential memory that is the size of the combined shape is allocated and the initial data is copied over.

Signal views cannot be merged in this manner because one has to assume that the whole signal base gets written to by another operator and it is not possible to cut out a small piece and move it to another memory location. Even if it were possible, this would contradict the purpose of the optimization algorithm as views are already parts of memory that are sequentially organized.

Consequently, views need to fulfill additional conditions to allow merging: their strides^1^ need to agree and they have to be sequential in memory. That is, the second view's offset has to be the first view's offset incremented by its size. To merge the views, no data is copied, but a new view encompassing both of the merged views is created.

### 3.3. Global effects of merging

When merging operators, a number of further global updates is required. Within the dependency graph all merged operators need to be removed and the new merged operator needs to be inserted. The dependencies of a new operator are given by the union of dependencies of the merged operators. Furthermore, when signals get merged, operators referencing these signals need to be updated. This is done by replacing the signal with a view into the merged signal.

### 3.4. Finding mergeable operators

A naive implementation would check whether each pair of operators could be merged. However, this would lead to a quadratic temporal scaling with the number of operators, which is undesirable. Thus, we make use of several restrictions about when it is possible to merge operators in order to improve the temporal scaling. Signal views place the most restrictions on which operators can be merged. As views with different bases can never be merged, we can consider groups of operators *O* where their first view shares the same base. (It is irrelevant how the associated signals are sorted for determining the first view as long as the sorting is consistent over all operators.) Operators without any views are considered to be their own group. The algorithm to find subsets of *O* that can be merged is given by the following:

**Require**: List *O* of operators. If any *o* ∈ *O* operates on a signal view, the bases of the first signal of each operator *o* need to be the same.

**Table:** 

1:	**procedure** perform_merges(*O*)
2:	*O* ← sort(*O*, sortkeys = [voffset(*o*) **for** *o* ∈ *O*])
	
3:	**for** *o*_*i*_ ∈ *O* **do**
4:	*m* ← [*o*]
5:	**for** *o*_*j*_ ∈ *O* with voffset(*o*_*i*_) + vsize(*o*_*i*_) ≤ voffset(*o*_*j*_) **do**
6:	**if** can_merge(*m*[−1], *o*_*j*_) **then**
7:	*m* ← append(*m, o*_*j*_)
8:	**else if** voffset(*m*[−1]) + vsize(*m*[−1]) < voffset(*o*_*j*_) **then**
9:	**break**
10:	**end if**
11:	**end for**
12:	**if** len(*m*) > 1 **then**
13:	merge(m)
14:	**else if** *o*_*i*_ uses a signal view **then**
15:	*O* ← *O* \ {*o*_*i*_}
16:	**end if**
17:	**end for**
18:	**end procedure**

In this *m*[−1] denotes the last element of the list *m*. Furthermore,voffset(*o*) and vsize(*o*) return the offset and size, respectively, of the first signal view associated with *o*. If an operator is not associated with any signal view, 0 is returned instead. This allows to use the same perform_merges(*O*) for operators with and without views. The algorithm first sorts the operators by the offset of their first signal view. Then for each operator, the sequence *m* of operators that can be merged is determined. This is essentially checked with the can_merge(*m*[−1], *o*_*j*_) function which evaluates the conditions described in Section 3.1.

The inner loop is cut short at the beginning (line 5) by skipping operators *o*_*j*_ that cannot be merged because the offset of the first view is lower than the end of the same view of *o*_*i*_. In other words, if *o*_*j*_ accesses items in a view that are in front of the items accessed by *o*_*i*_, the operators cannot be merged because of non-sequential memory access, and these cases are skipped. The loop is cut short at the end in a similar way in line 8 when all further operators cannot be merged to the current set *m* because their views are not consecutive anymore. If none of the associated signals of the operators in *O* is a view, those statements will not have any effect as voffset(*o*) and vsize(*o*) return 0. In that case all combinations of *o*_*i*_, *o*_*j*_ ∈ *O, o*_*i*_ ≠ *o*_*j*_ have to be considered.

Furthermore, line 15 excludes operators that can never be merged with any other operator from future optimization passes. If an operator with a view cannot be merged with any other operator in a single optimization pass, this will not change in later optimization passes. If the views themselves are incompatible for merging, they will stay incompatible because signal views only change through merging. If other non-view signals are incompatible, these might become signal views with further merges, but this will not influence the compatibility for merging as the data type and the shape (except for the concatenation axis) do not change. Lastly, if the operator cannot be merged because of dependency relationships, these will not be changed by merging of other operators either.

### 3.5. Pre-grouping operators

It is possible to just group operators by the base of the first view and then to pass them to perform_merges(*O*). It is, however, more efficient to group them by the operator type first, since different operator types can never be merged. Furthermore, depending on which operator types are merged first, the final computation graph will be different, and this can effect the final efficiency. Consequently, we start with merging ElementwiseInc, Copy, DotInc, SimNeurons (in this order) before merging other operator types in an undefined order. This order is a heuristic determined by trial and error that outperforms other orders in most cases.

**Figure 3:**
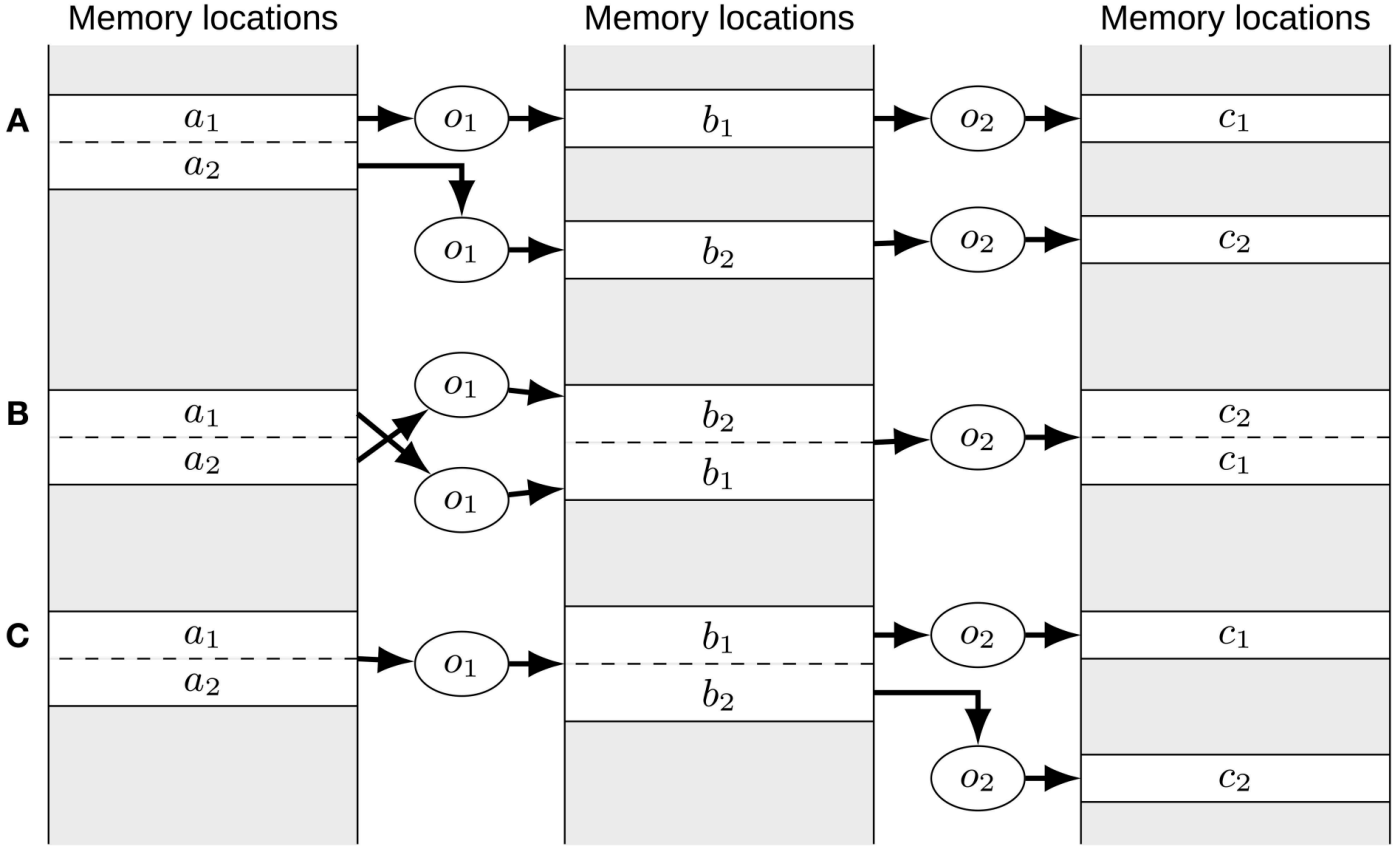
**The order of merge operations can matter. (A)** Initial situation. The *o*_1_ operators access different views of the *a* signal. All other signals are independent. If the *o*_2_ operators are merged first, no order is imposed on the *b* and *c* signals except that the order of the *b* and *c* signals need to correspond. As such (*b*_2_, *b*_1_) and (*c*_2_, *c*_1_) might be chosen as the order, yielding the situation depicted in **(B)**. Here, it is not possible to merge the *o*_1_ operators as the order of the *a* and *b* signals is swapped in memory. This can be avoided by merging operators accessing views first as done in **(C**). Here, the *o*_1_ operators need to be merged first which will impose the order of the *a* signal views onto the *b* signals. Then it is still possible to also merge the *o*_2_ operators as now the order of the *b* signals can be imposed on the *c* signals.

Notably, merges for one operator type might make merges for other operator types possible. Thus, we do multiple passes over merges of all operator types until no further reduction in the number of operators is obtained. In these passes we disallow the merging of operators by default that do not reference any views. The rationale for this is that pure signals do not impose any order on the merged operators. Thus, the order might not correspond to the order imposed on other operators preventing further merges. This is illustrated in Figure 3. When no further reduction in the number of operators is possible, only the merging of operators without a view reference is allowed for one pass. In addition, we stop the optimization process once the number of merged operators per second in one pass falls below one percent of the average over all passes. This prevents the optimization process from taking excessively long if only small further reductions in the operator number are possible. The following pseudo-code makes this process explicit:

**Require** List *O* of all operators.

**Table:** 

1:	*n*_all_ ← |*O*|, *n*_0_ ← |*O*|, *n*_1_ ← 0
2:	*t*_start_ ← time, *t*_pass_ ← time
3:	*v* ← **true**
4:	**while** *v* **or** *n*_1_ < *n*_0_ and $0.01\frac{n_{\text{all}}}{\text{TIME}-t_{\text{start}}}<\frac{n_{0}-n_{1}}{\text{TIME}-t_{\text{pass}}}$ **do**
5:	*t*_pass_ ← time
6:	*v* ← *n*_0_ ≠ *n*_1_
7:	Ô ← [*o* **for** *o* ∈ *O* **if** has_view(*o*) = *v*]
	
8:	*n*_0_ ← |*O*|
9:	**for** *O*_*k*_ ∈ group_operators(Ô) **do**
10:	perform_merges(*O*_*k*_)
11:	**end for**
12:	*n*_1_ ← |*O*|
13:	**end while**

The variables *n*_0_ and *n*_1_ are used to compare the number of operators |*O*| before and after an optimization pass; *v* indicates whether the merging of operators without associated views is allowed in the optimization pass. The time function retrieves the current time. Whether an operator has an associated signal view is checked with the has_view(*o*) function. The group_operators(Ô) function is used to group operators by their type and return a list of lists of single operator types. The return value is sorted according to the aforementioned heuristic.

### 3.6. Transitive closure of the dependency graph

To check whether one operator depends on another operator we use the transitive closure of the dependency graph. In the transitive closure of a graph, one adds edges (*v*_*i*_, *v*_*j*_) for each vertex *v*_*j*_ that is reachable (in any number of steps) from vertex *v*_*i*_. Thus, it enables checking whether one vertex, or operator in this case, depends on another in amortized constant time with the usage of hash-tables to store the edges of each vertex.

Some further care in the representation of this transitive closure graph has to be taken. Because of the large number of operators in many Nengo models, an adjacency matrix would require too much memory as it size increase quadratically. Storing the edges for each vertex in a hash-table would still require too much memory as most nodes will have many edges in the transitive closure. Luckily, due to the structure of the graph generated by Nengo, many operators share the same transitive closure. In other words, many vertices in the transitive closure of a typical Nengo dependency graph will have exactly the same set of outgoing edges. Thus, we can significantly reduce the memory consumption by hashing each set of edges and reuse the same set instance where appropriate instead of creating multiple set instances representing the same information.

## 4. Results

To demonstrate effect of the optimization of the computation graph, we ran benchmarks on three different neural models ranging from a small toy example to a very large scale model. In all cases the optimizations provides a significant speed up of the simulation times that will exceed the additional build time in most cases. The source code to run the benchmarks is available at https://github.com/ctn-archive/gosmann-frontiers2017.

Benchmarks followed the same protocol as in Bekolay et al. (2014). For each trial the time required to build the model was recorded as the build time, then 10 simulation time steps were run to pre-fill memory buffers, and finally the model was simulated for 1,000 time steps corresponding to one second of simulated time. The time required to simulate those 1,000 time steps is reported as simulation time. The simulation times stated in seconds can also be interpreted as the factor by which the model runs slower than real time. The benchmarking protocol gave a very low variability of measured times. For each benchmarking condition, five trials were averaged.

Besides the Nengo reference backend and the optimized (reference) backend, we ran the same benchmarks with Nengo-OCL, the OpenCL implementation of Nengo, on CPU and GPU. All benchmarks were run on a computer with two 4-core Intel Xeon E5540 2.53 GHz CPUs and an Nvidia Tesla C2050 GPU. On the software side Python 3.4.2 with NumPy 1.12.0 on Debian Linux 8 was used. To achieve optimal performance, NumPy was linked against a version of OpenBLAS compiled for the specific machine.

The Nengo simulator supports a variety of different neurons models. Here we focus on the spiking leaky integrate-and-fire (LIF) model, a simple model that captures the spiking behavior of cortical neurons. The results are similar with rate-based LIF neurons but are omitted for brevity. In addition, we report the *direct mode* simulation results, where desired functions are computed exactly without the use of individual neurons. This mode is usually used for debugging because it eliminates neuron noise and because it is faster for the reference backend, although behavioral results can deviate significantly.

### 4.1. Circular convolution network

The operation of circular convolution, defined as

(2) $$u=v⊛w:\;u_{i}=\sum_{j\;=\;1}^{n}v_{j}w_{(i-j)\;\mathrm{mod}\;n}\text{,}$$

is used in many Nengo models of cognitive tasks based on the Semantic Pointer Architecture (SPA; Eliasmith, 2013). Within the SPA, circular convolution is used to bind and unbind different concepts, represented by high-dimensional vectors.

**Figure 4:**
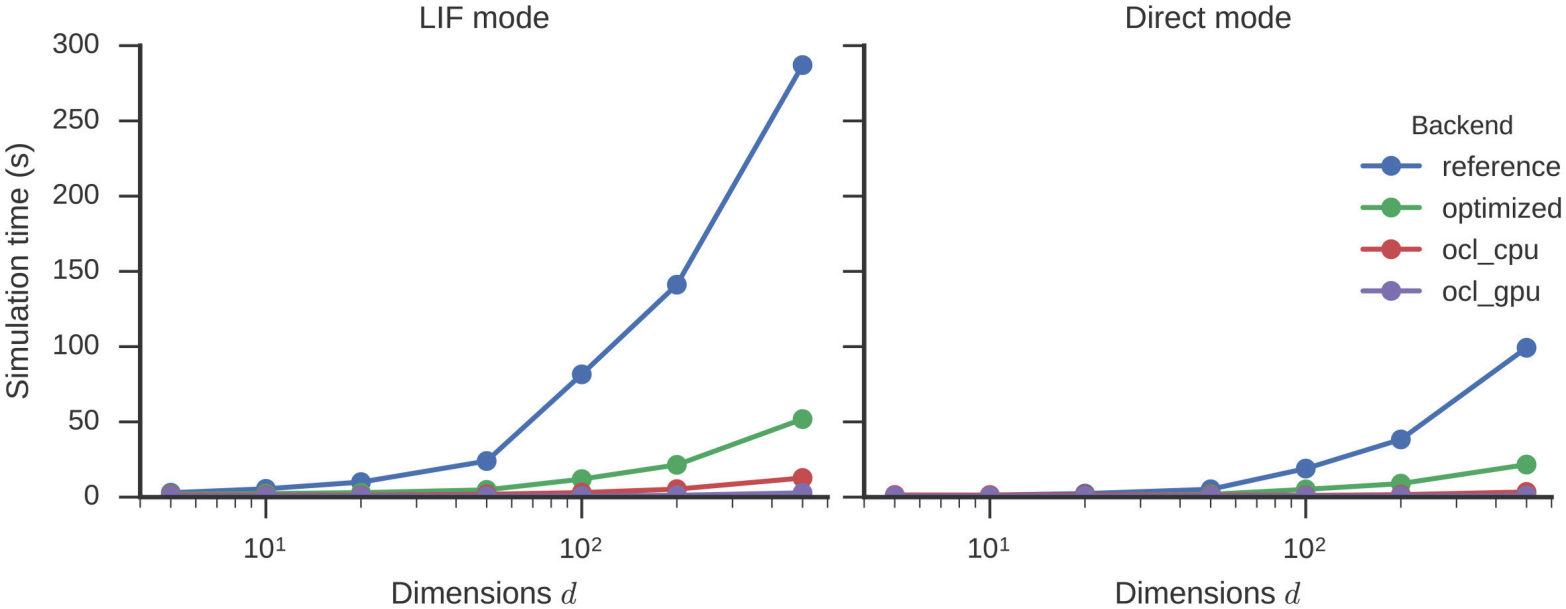
**Time required to simulate a circular convolution network of different dimensionalities with 500 neurons per dimension for one second**. Error bars show bootstrapped 95% confidence intervals but are small enough to be hidden behind the data point markers. Data were collected on four Nengo backends: the reference backend (reference), the reference backend with the optimizations described here (optimized), Nengo-OCL using the CPU (ocl_cpu), and Nengo-OCL using the GPU (ocl_gpu).

The simulation time of circular convolutions with vectors of different dimensionality and 500 neurons for each dimension is shown in Figure 4. Higher dimensionalities require more neurons to be simulated which leads to an increase in simulation time. With the optimizations enabled, this increase is less steep. Simulating a 500-dimensional circular convolution for 1 s takes 287 s with the reference backend, but only 52 s with the optimizations (5.5 times speed-up). The highest speed-up, with a factor of 6.8, is achieved for 100 dimensions. This optimized neural simulation takes less time than the reference backend in direct mode. For 500 dimensions, for example, 99 s are taken by the reference backend in direct mode while the optimized backend only spends 52 s on simulating LIF neurons or 22 s when in direct mode.

**Figure 5:**
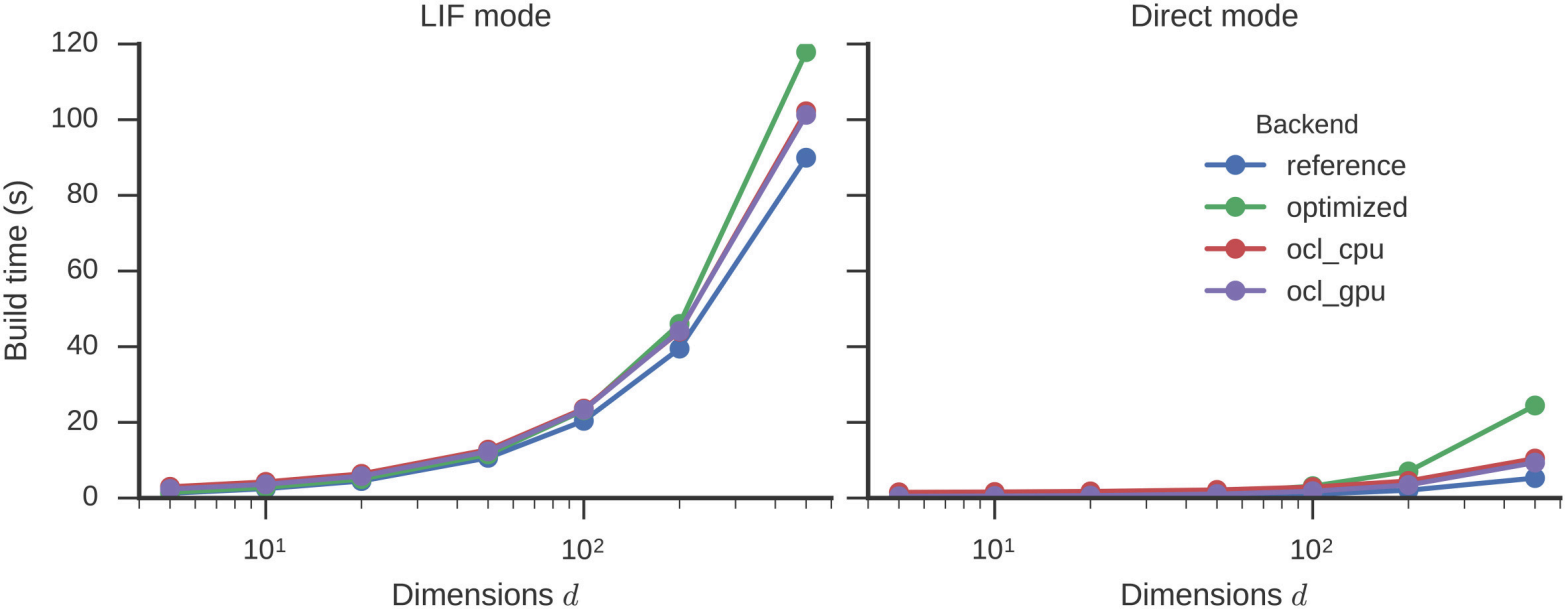
**Time required to build a circular convolution network of different dimensionalities with 500 neurons per dimension**. Error bars show bootstrapped 95% confidence intervals but are small enough to be hidden behind the data point markers. Data were collected on four Nengo backends: the reference backend (reference), the reference backend with the optimizations described here (optimized), Nengo-OCL using the CPU (ocl_cpu), and Nengo OCL using the GPU (ocl_gpu).

The optimizations increase the build time (Figure 5) only marginally: by a factor of 1.3 (90 to 118 s) for 500 dimensions with LIF neurons. The OCL backend on both CPU and GPU is fastest in terms of simulations times and requires build times in-between the reference and optimized backend. The optimization decreases the number of operators in the graph from 26,167 to 85 for 500 dimensions.

### 4.2. N-back task model

The n-back task is used in psychological research as a test of working memory and its maintenance. In this task a subject is sequentially presented with words or spatial locations and has to indicate repetitions that are exactly *n* positions apart (e.g., by a button press). Gosmann and Eliasmith (2015) presented a medium sized Nengo model performing this task with 92,250 neurons. Here we use this model as a benchmark. Items that have to be remembered are represented as *d*-dimensional vectors with *d* = 64 in this model. Neural groups within the model, however, do not represent the full *d*-dimensional vectors, but *s* groups representing *d*/*s* dimensional parts of the vectors are combined. This is done for three reasons. First, to achieve the same accuracy, a larger number of neurons would be required to represent the full dimensionality in one large group of neurons than when representing it in pieces in several smaller groups. Second, the decoder computation requires an *O*(*N*^3^) matrix inversion where *N* is the number of neurons in a single group of neurons, whereas with *s* smaller groups of *N*/*s* neurons it is *O*(*N*^3^/*s*^2^). Third, as noted in Section 1, optimizations to achieve an improved representational accuracy (Gosmann and Eliasmith, 2016) work better if the vector is split into smaller pieces. A disadvantage of representing small parts of a vector is that the total number of neural groups increases, which produces a larger number of operators in the computation graph that have to be iterated over during the simulation. Thus, the simulation time increases. Here, we look at the performance when using *s* = 64 and *s* = 4 splits.

**Figure 6:**
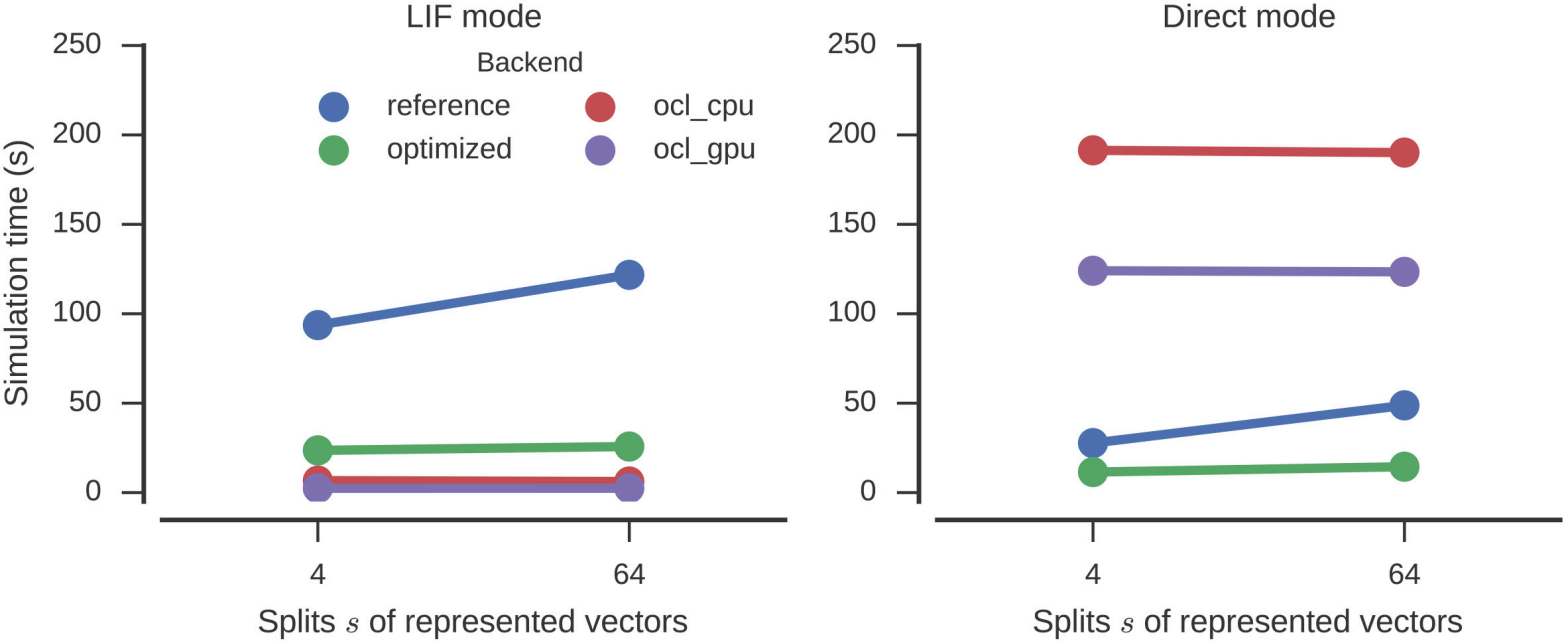
**Time required to simulate the n-back model with different numbers of splits of the represented vectors for one second**. Error bars show bootstrapped 95% confidence intervals but are small enough to be hidden behind the markers. Data were collected on four Nengo backends: the reference backend (reference), the reference backend with optimizations (optimized), Nengo-OCL using the CPU (ocl_cpu), and Nengo-OCL using the GPU (ocl_gpu).

For LIF neurons, the results are similar to the circular convolution (Figure 6). The simulation time is decreased by a factor of 4.7 and 4.0 for 64 and 4 splits, respectively, (121 to 26 s and 94 to 24 s) by the optimizations. This again surpasses the direct mode speed of the reference backend (49 and 28 s). Again Nengo-OCL is faster (less than 7 s in all conditions except direct mode), but in direct mode it ends up being considerably slower (at least 124 s).

**Figure 7:**
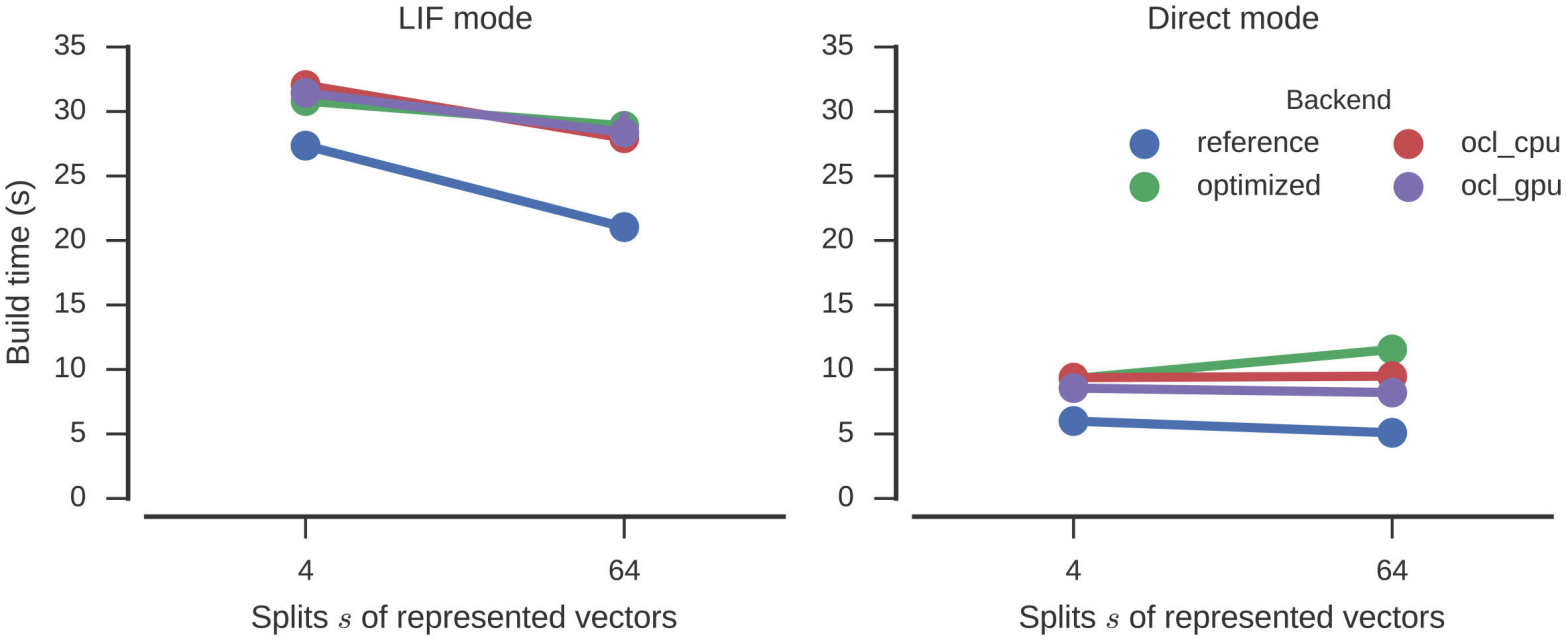
**Time required to build the n-back model with different numbers of splits of the represented vectors**. Error bars show bootstrapped 95% confidence intervals but are small enough to be hidden behind the markers. Data were collected on four Nengo backends: the reference backend (reference), the reference backend with optimizations (optimized), Nengo-OCL using the CPU (ocl_cpu), and Nengo-OCL using the GPU (ocl_gpu).

The build times (Figure 7) are slightly increased by the optimizations, but about the same as for Nengo-OCL. The number of operators is decreased from 14,905 to 1,135 (*s* = 64) and from 6,265 to 407 (*s* = 4).

### 4.3. Spaun

Our final benchmark uses an updated version of the Spaun model (Eliasmith et al., 2012). With originally 2.5 million and almost 4 million neurons in the version used here, it is the largest functional brain model reported to date. Thus, it is a good proxy for the largest models that are currently run with Nengo. The Spaun model can perform eight different cognitive tasks, such as list learning or copy drawing. It gets input through 28 by 28 pixel images and produces output with a simulated arm.

**Figure 8:**
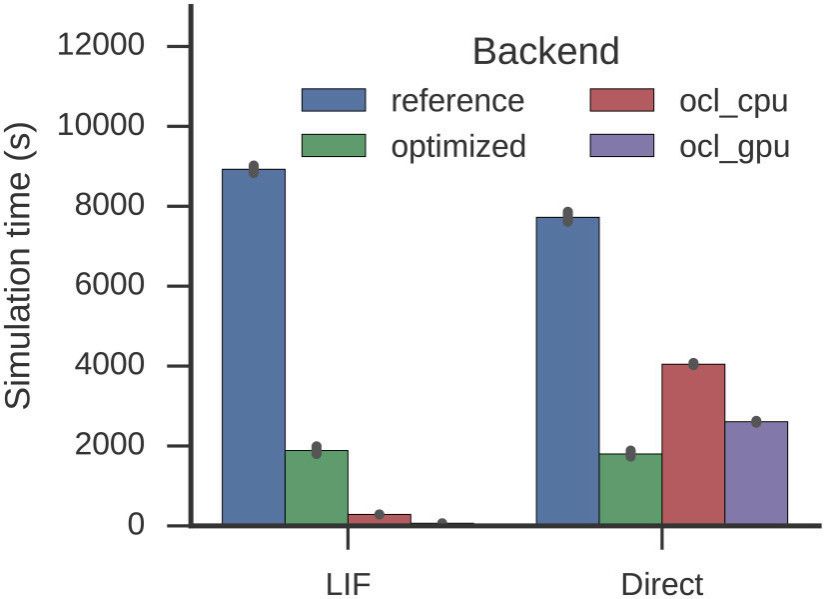
**Time required to simulate the Spaun model for one second**. Error bars show bootstrapped 95% confidence intervals. Data were collected on four Nengo backends: the reference backend (reference), the reference backend with the optimizations described here (optimized), Nengo-OCL using the CPU (ocl_cpu), and Nengo-OCL using the GPU (ocl_gpu).

The optimization reduces the time required to simulate one second from 8,926 to 1,886 s (factor 4.7 speed-up, Figure 8). This is similar for the direct mode (7,723 s to 1,800 s equivalent to a factor 4.3 speed-up). Again, with the optimizations LIF neurons are barely more expensive to simulate than the direct mode. Nengo-OCL takes 286 s to simulate one second on the CPU and 66 s to simulate one second on the GPU, but for direct mode it takes with 4,046 and 2,607 s, respectively, longer than the optimized backend.

**Figure 9:**
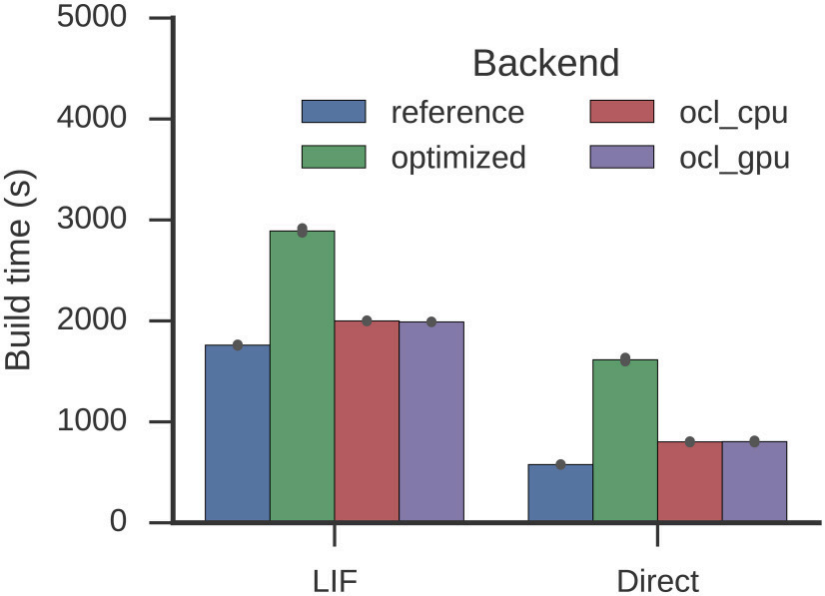
**Time required to build the Spaun model**. Error bars show bootstrapped 95% confidence intervals. Data were collected on four Nengo backends: the reference backend (reference), the reference backend with the optimizations described here (optimized), Nengo-OCL using the CPU (ocl_cpu), and Nengo-OCL using the GPU (ocl_gpu).

For Spaun, build times are increased to 2,891 from 1,760 s (LIF) and to 1,615 from 577 s (direct mode) which also exceeds the Nengo-OCL build times around 2,000 and 800 s (Figure 9). The optimization process reduces 996,917 to 161,161 operators.

### 4.4. Memory usage

While simulation time is our main concern in this work, the memory usage is of some importance as well. In fact, some care has to be taken in the representation of the transitive closure graph (see Section 3.6) to ensure reasonable memory usage. With this, however, memory usage is within reasonable bounds. To verify this, we ran the same benchmark models as before and recorded the maximum unique set size as an indication of the memory usage. The unique set size is the amount of memory that is private to a process and not shared with other processes.

With the optimizer, the maximum memory usage increased to 420 MiB from 284 MiB for the circular convolution of 500 dimensions with 500 neurons per dimension. Surprisingly, the maximum memory usage decreased to 216 MiB from 223 MiB for the n-back model. This is most likely due to variability introduced by the Python memory allocation mechanisms. Finally, the maximum memory usage for the Spaun model increased to 31 GiB from 12 GiB. This is a large difference in absolute terms, but not unreasonable for a model of that size.

## 5. Discussion

We presented an algorithm to improve the simulation speed of the Nengo neural network simulator. This is achieved by reducing the number of operations in the computational graph generated from the model description and allocating manipulated data in consecutive memory blocks that better utilize CPU pre-fetching and caching. We found a speed-up over a wide range of model sizes and complexities. For complex models the speed-up is typically around 4 times, but can be less for very small models. The highest speed-up of 6.8 was obtained for a circular convolution with 100 dimensions. Nevertheless, the Nengo-OCL backend is still an additional order of magnitude faster. This is not surprising as GPUs, due to their specialized nature, can be powerful for linear algebra applications. As well, on the CPU the OCL implementation allows the simulator to utilize all CPU cores to the best extent. The Python implementation of the reference backend is limited in this regard. The main Python loop is single threaded and only the NumPy function calls can make use of multiple cores to the extent it is supported by the respective underlying linear algebra library. Because of Python's global interpreter lock (GIL), a multi-threaded implementation would not provide any gain in efficiency. Alternatively, one could use multi-processing to circumvent the GIL, but would then lose efficiency due to the communication overhead between different processes. Thus, the execution of multiple operators cannot be parallelized in an efficient manner in a pure Python implementation which prevents the application of many methods from dataflow programming that focus on scheduling a computation graph for parallel execution (e.g., Reiter, 1968; Miller, 1973; Hendrickson and Leland, 1995). If we were to drop the requirement of a pure Python implementation, it might be worthwhile to investigate whether further speed-ups for the reference backend can be obtained with Cython (Behnel et al., 2011) to release the GIL and parallelize the operator execution. But it is not clear if this achieves a performance close to other non-pure Python backends.

Given faster Nengo backends, one might ask whether these optimizations to the reference backend are still worthwhile. We believe that this is the case for several reasons. Most importantly, not everyone is able to run Nengo-OCL because GPUs are a cost in addition to the CPU and Nengo-OCL does not support execution on CPUs on all platforms. Also, the installation of Nengo-OCL is non-trivial on some platforms. Furthermore, the optimizer allows modelers to use all features implemented in the reference backend. Maintainers of other backends, including Nengo-OCL, can decide to not implement a certain set of features or only do so with a delay (only the reference backend is currently feature complete). Similarly, it is easier to prototype and test new features with a pure Python implementation in the reference backend than developing specialized C code for the Nengo-OCL backend. Finally, with the optimizations suggested here, the reference backend is faster in direct mode than Nengo-OCL except for very simple models. This can be explained by the fact that Nengo-OCL cannot run arbitrary Python code on its computing device and all functions applied to connections between neural groups need to run some arbitrary Python code in direct mode. While one of the main reasons to use direct mode was simulation speed, it can also be helpful in debugging models as it provides exact mathematical solutions instead of neural approximations. In these debugging cases using the optimized reference backend provides faster simulations than Nengo-OCL.

An increase in simulation speed would not be of much use if it were to largely increase the model build times. Fortunately, this is not the case. While build times increase moderately, the gain in simulation speed will be larger in most cases. For example, the increased build time for the Spaun model is completely offset by the savings in simulation time for simulated durations of at least 0.16 s.

It is also worth highlighting synergies of this optimization with methods presented by Gosmann and Eliasmith (2016). For Nengo models employing the Semantic Pointer Architecture (SPA), which for example the n-back task model and Spaun do, it is possible to optimize the neural representations to require fewer neurons while keeping the representational error constant. A greater reduction in neuron number can be achieved by splitting the dimensions of a high-dimensional unit-vector (a Semantic Pointer) into individual groups of neurons. This changes the distribution of values that needs to be represented by each group of neurons from a uniform distribution to a skewed distribution. By optimizing the decoders only for frequent values in that skewed distribution, the same accuracy can be achieved with fewer neurons in total. One might assume that fewer neurons leads to a decrease in simulation time, but the total number of neural groups in the model increases. This increases the number of operators in the generated computation graph, increasing the simulation time. The merging of operators through the optimization, while potentially not completely eliminating this increase, counteracts much of it. Thus, combining both optimization methods makes models more efficient to simulate.

In the near future we expect to integrate the described optimization into Nengo for inclusion in one of the upcoming releases. Furthermore, we intend to investigate how much other Nengo backends, like Nengo-OCL, can benefit from similar optimization methods. We also believe that this sort of optimization might be beneficial to other software packages using computation graphs, most prominently Theano and Tensorflow which are widely used in the deep learning community.

## Author contributions

Conceived and implemented the algorithm: JG. Ran and evaluated the benchmarks: JG. Wrote the paper: JG, CE.

## Funding

This work has been supported by the Canada Research Chairs program, the NSERC Discovery grant 261453, Air Force Office of Scientific Research grant FA8655-13-1-3084, CFI, and OIT.

### Conflict of interest statement

The authors declare that the research was conducted during or after a financial relationship with Applied Brain Research, Inc. which determines the licensing for Nengo, which is free for personal and research purposes.

## Footnotes

^1^The strides define the number of bytes to skip to move along a certain array axis.
